# Sudden Death Due to Hypercoagulability in a Patient With Pancreatic Cancer and Diabetic Ketoacidosis

**DOI:** 10.7759/cureus.83152

**Published:** 2025-04-28

**Authors:** Esther Park, Milenko T Petrovic, Nidal Shah, Rahul Sharma

**Affiliations:** 1 Pathology, University of Arkansas for Medical Sciences, Little Rock, USA; 2 Internal Medicine, University of Arkansas for Medical Sciences, Little Rock, USA

**Keywords:** acute pulmonary embolism, code status, diabetic ketoacidosis (dka), endoscopy, fatal outcome, malignancy-associated hypercoagulability, pancreatic cancer, type 2 diabetes mellitus

## Abstract

Pancreatic cancer is a significant contributor to cancer-related mortality, with an increasing incidence linked to an aging population. A rare complication of pancreatic cancer is diabetic ketoacidosis (DKA), which arises from the tumor’s impairment of insulin production. DKA can also present alongside other challenges such as malnutrition and hypercoagulability. This study describes a 67-year-old female patient with a past medical history of type 2 diabetes who presented with DKA and was later diagnosed with a pancreatic mass suggestive of malignancy. She developed multiple venous thrombi and subsequent pulmonary emboli, leading to sudden death. Autopsy revealed extensive occlusive thrombi and ischemic changes, emphasizing the potentially life-threatening interactions between DKA, malignancy-associated hypercoagulability, and metabolic derangements of pancreatic cancer. This study reinforces the need for careful monitoring and management of thrombotic events in patients with multiple comorbidities.

## Introduction

Pancreatic cancer is a leading cause of cancer-related mortality, with a prevalence that has doubled over the past 25 years [1]. As the United States population ages, its incidence has risen [2]. A rare but severe complication in the context of pancreatic cancer is diabetic ketoacidosis (DKA), an acute and life-threatening complication of diabetes [3,4]. DKA is not only linked to long-standing diabetes but also inducible by pancreatic tumors disrupting insulin production, leading to pancreatogenic diabetes mellitus. Compromise of the pancreas can result in pancreatogenic diabetes mellitus, which arises due to destruction of insulin-producing islet cells [5]. DKA in the setting of pancreatic cancer presents unique challenges, since patients can suffer from malnutrition, dehydration, cachexia, and hypercoagulability [6,7]. Pancreatic adenocarcinomas also predispose patients to hypercoagulability, increasing venous thromboembolism (VTE) risk due to tumor-derived procoagulant factors [8]. This case examines the sudden death of a patient with pancreatic cancer and DKA secondary to hypercoagulation. In addition, this study functions to remind clinicians of the importance of early recognition for management and outcomes of patients with pancreatic cancer.

## Case presentation

This 67-year-old female with a history of type 2 diabetes mellitus, hypertension, binge-eating disorder, hypothyroidism, and laparoscopic cholecystectomy presented to an outside hospital with abdominal pain, confusion, nausea, and vomiting. She was diagnosed with DKA and a urinary tract infection (UTI) leading to sepsis, treated with an insulin drip, fluids, and ceftriaxone. Labs were obtained at this time to rule out sepsis, PE, and cardiac causes. These values are summarized in Table 1.

**Table 1:** Laboratory data obtained from an outside hospital at which the patient initially presented. These parameters support the diagnosis of diabetic ketoacidosis and urinary tract infection.

Lab parameters	Value	Reference ranges
Glucose	468 mg/dL	70-110 mg/dL
WBC	16.65 K/µL	3.60-9.50 K/µL
Lactate	3.5 U/L	0.5-2.2 U/L
Bacteria, urine	Positive	None/HPF
Leukocyte esterase, urine	Positive	0-5/HPF

She was transferred to the University of Arkansas for Medical Sciences (UAMS) Medical Intensive Care Unit (MICU) for advanced care. Upon admission, the patient's DKA resolved, and she was transitioned to 10 units of glargine with a sliding scale insulin regimen targeting blood glucose below 180 mg/dL. The next day, she experienced two episodes of coffee-ground emesis. An esophagogastroduodenoscopy (EGD) two days later revealed grade D esophagitis. The patient’s labs obtained following transfer to UAMS are summarized in Table 2.

**Table 2:** Laboratory values taken at UAMS. These labs paint a more complete clinical picture of this patient with multiple comorbidities. Of note are the patient's glycosuria, ketonuria, and elevated troponin. WBC: white blood cell; CA 19-9: carbohydrate antigen 19-9; AST: aspartate aminotransferase; ALT: alanine aminotransferase; GGT: gamma-glutamyl transferase; TSH: thyroid-stimulating hormone; UAMS: University of Arkansas for Medical Sciences

Lab parameters	Value	Reference ranges
Glucose	289 mg/dL	70-110 mg/dL
WBC	19.16 K/µL	3.60-9.50 K/µL
Lipase	25 U/L	13-60 U/L
Amylase	36 U/L	28-100 U/L
CA 19-9	<0.8 U/mL	0.0-37.0 U/mL
Lactate	2.4 mmol/L	0.5-2.2 mmol/L
Beta-hydroxybutyrate	0.90 mmol/L	≤0.27 mmol/L
Troponin	446.9 ng/L	<14.9 ng/L
Alkaline phosphatase	199 IU/L	32-91 IU/L
AST	84 IU/L	15-41 IU/L
ALT	39 IU/L	4-45 IU/L
Lactate dehydrogenase	514 IU/L	100-248 IU/L
GGT	120 IU/L	7-50 IU/L
TSH	6.50 µIU/mL	0.34-5.60 µIU/mL
T4, free	0.55 ng/dL	0.58-1.64 ng/dL
Glucose, urine	>1000 mg/dL	0-15 mg/dL
Ketones, urine	40 mg/dL	None-to-trace
Bacteria, urine	Rare	None/HPF
WBC, urine	3-5/HPF	0-2/HPF
Hyaline casts, urine	Present	0-2/HPF

**Figure 1:**
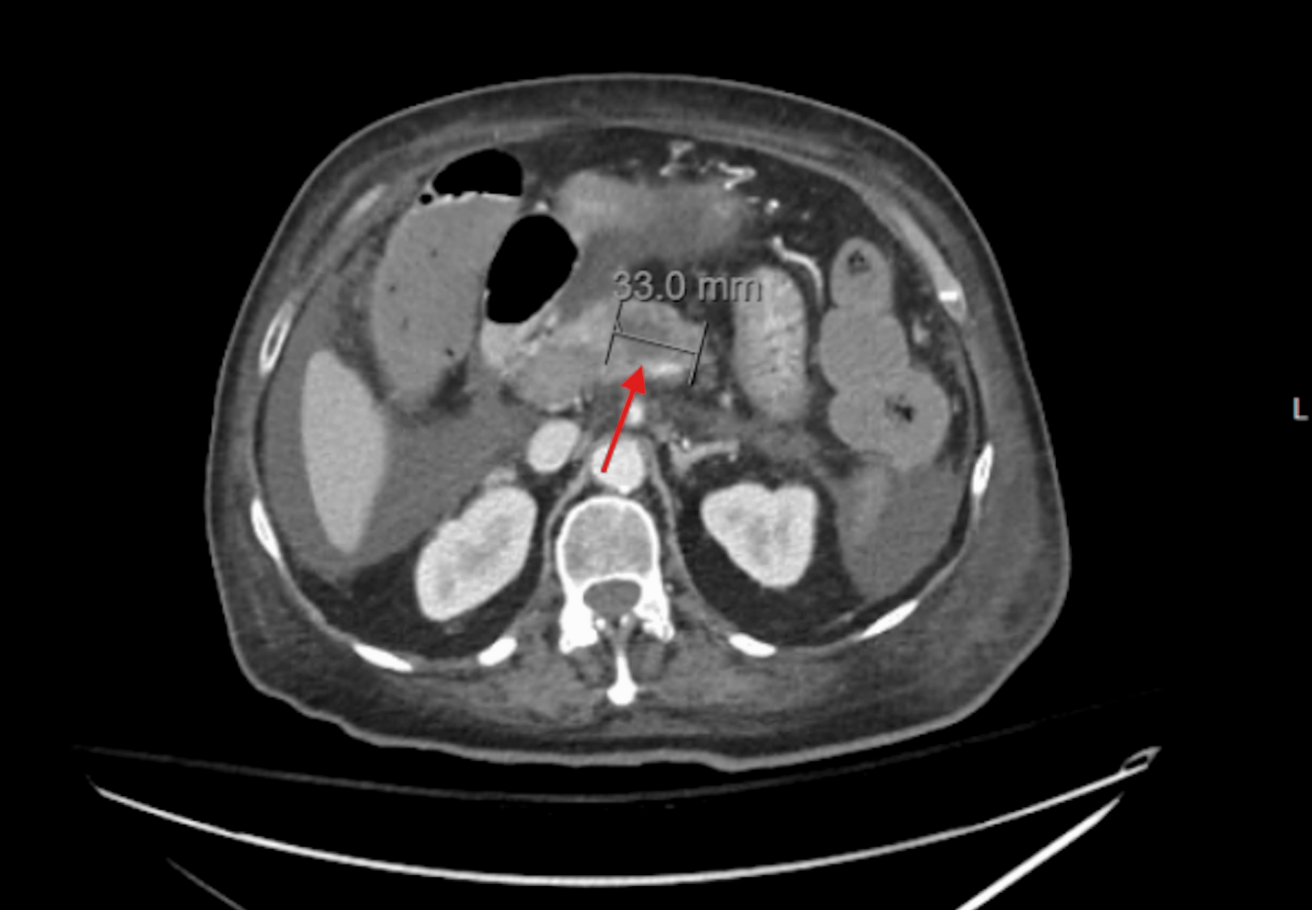
MRI image of hypoenhancing pancreatic mass in the proximal body of the pancreas. The arrow points to the mass and the line indicates its size.

This troponin elevation suggests myocardial strain or injury, likely related to the recent episode of sepsis or right heart strain from PE. Some time after, her oxygen saturation dropped to the low 70s (%) on 6 L/min of nasal cannula oxygen. Despite efforts to encourage deep breathing, there was no improvement in her oxygen saturation. Increasing the flow to 15 L/min improved saturation to 95%. A subsequent MRI showed a 33.0 mm hypoenhancing necrotic pancreatic mass in the proximal body, suggestive of malignancy, with acute thrombi in the superior mesenteric vein, portal vein, and splenic vein (Figure 1).

**Figure 2:**
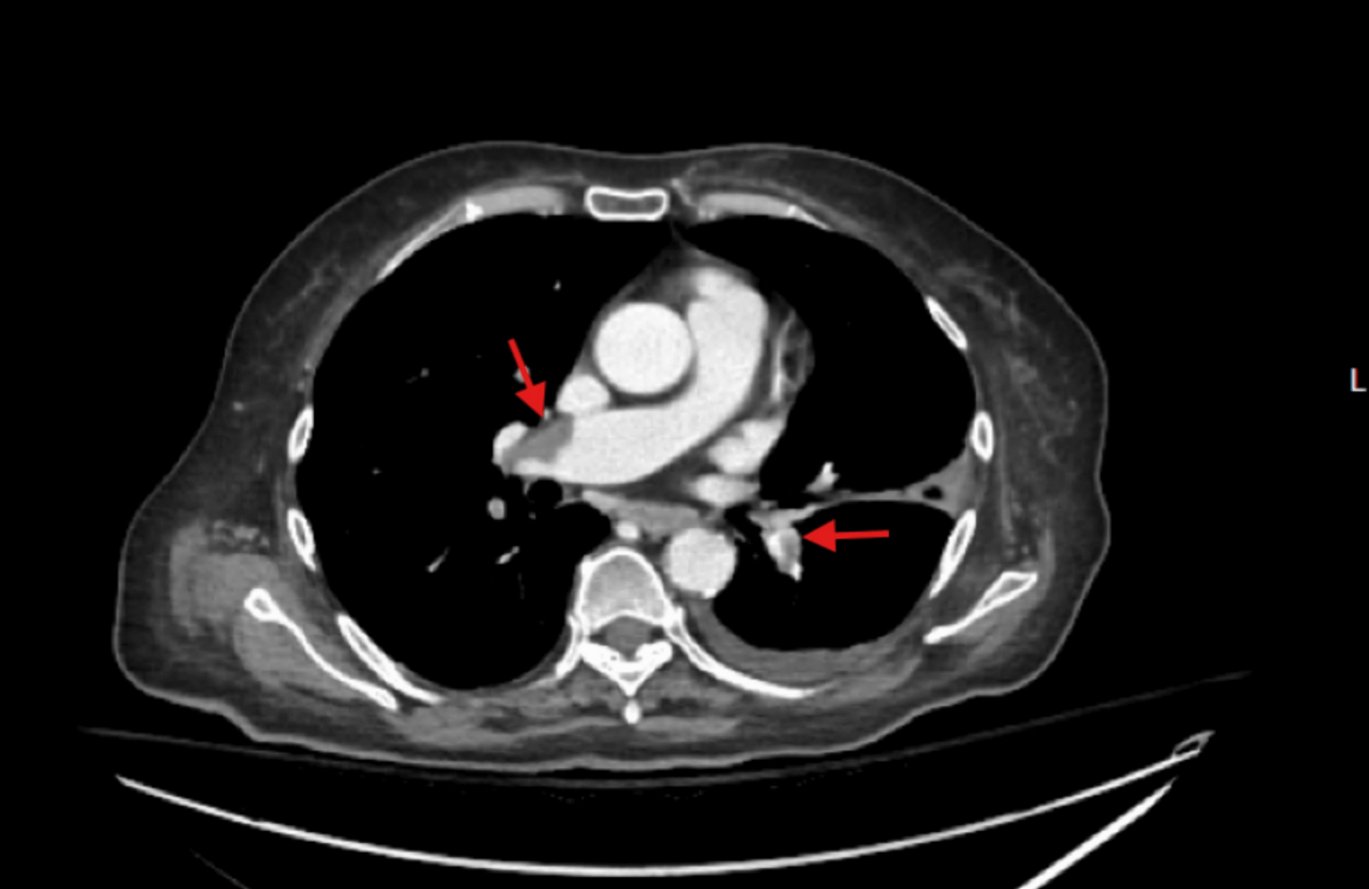
CT image showing bilateral pulmonary emboli (arrows). This patient had systemic thrombi that embolized to the pulmonary system.

The patient was started on a heparin drip for anticoagulation in anticipation of endoscopic ultrasound (EUS). A CT one day later confirmed bilateral pulmonary emboli, portal vein thrombi extending to splenic and superior mesenteric veins, and severe celiac artery stenosis (Figure 2). In addition, diffuse wall thickening and submucosal edema of small bowel loops were noted, raising concern for bowel ischemia.

Five days after she first presented to UAMS, EUS was performed with fine needle aspiration biopsy for tissue diagnosis. During the procedure, the patient became hypoxic, required intubation, and coded shortly after. Despite CPR, epinephrine, defibrillation, and tissue plasminogen activator (tPA), she could not be revived and was declared dead at 10:26 am. The patient’s family requested an autopsy.

Autopsy revealed an occlusive thrombus in the pulmonary trunk as well as bilateral pleural effusions of 450 mL serous fluid. Multiple venous thrombi were noted, namely an occlusive mesenteric thrombus and an accompanying segment of small bowel with ischemic changes. In addition, a portal vein thrombus was observed extending into the superior vena cava and splenic vein. Her pancreatic mass measured at 7.5 cm. Ulcerative esophagitis and ascites (2.5 L) were also observed.

## Discussion

This 67-year-old woman presented with DKA and a clinical course marked by metabolic and thrombotic challenges. Malnutrition, dehydration, and ischemic bowel changes further complicated her condition. Imaging confirmed a pancreatic malignancy with extensive venous thromboembolism (VTE), consistent with cancer-associated hypercoagulability (Trousseau’s syndrome). Initial laboratory workup, as detailed in Tables 1, 2, revealed glycosuria and ketonuria consistent with DKA, as well as other elevated parameters such as lactate dehydrogenase, troponin, and gamma-glutamyl transferase (Table 2). The patient’s elevated lactate supported the diagnosis of sepsis, as did her leukocytosis. The presence of hyaline casts suggests dehydration as well. Furthermore, elevated troponin levels can be seen in the context of sepsis and have been associated with increased mortality in cases of both PE and sepsis [9,10].

The patient’s sudden death during EUS highlights the lethal interplay of DKA, malignancy, and thrombosis. There were multiple risk factors that could have precipitated the development of diabetic ketoacidosis, including malignancy, UTI, and poorly controlled diabetes. While DKA is more commonly associated with type 1 diabetes, pancreatic cancer has in some cases been reported to cause this state [5,11]. DKA can arise from pancreatogenic diabetes as tumors destroy insulin-producing islet cells, impairing glucose regulation. This has occasionally been reported in the literature. In addition, DKA treatment can independently induce a prothrombotic state, amplifying cancer-related risks [12]. The patient’s UTI likely exacerbated this by causing dehydration and catecholamine-associated insulin resistance [13]. Her type 2 diabetes history also suggests possible medication non-adherence, though unconfirmed.

Pancreatic adenocarcinoma has historically been strongly linked to hypercoagulability via procoagulant factors like tissue factor and inflammatory cytokines [8]. This patient exhibited multiple thrombi (superior mesenteric vein, portal vein, splenic vein, and pulmonary arteries), refractory to tPA during her final event. She likely had Trousseau’s syndrome, or cancer-associated thrombosis, in which the risk of VTE in malignancy is heightened due to tumor-related factors such as increased secretion of tissue factor and inflammatory cytokines [14,15]. Despite anticoagulation therapy, patients with pancreatic cancer often have recurrent and refractory thrombosis due to the persistent prothrombotic environment [16]. Autopsy confirmed an occlusive pulmonary trunk thrombus as the cause of death, compounded by bilateral pulmonary emboli evident on prior CT.

The patient was likely already vulnerable to episodes of respiratory desaturation due to her bilateral pulmonary emboli. Indeed, a particularly dangerous complication in this patient was the development of extensive VTE. Earlier VTE prophylaxis might have been considered, though her bleeding risk (esophagitis, emesis) posed a challenge.

Despite stabilization efforts, her death underscores the difficulty of managing pancreatic cancer with overlapping acute complications. The patient succumbed to bilateral pulmonary emboli, which were confirmed on autopsy. Finally, the hypoxia during EUS may suggest a procedure-related embolic event, though this is speculative, emphasizing procedural risks in such patients. The above events highlight the need for vigilant monitoring and multidisciplinary care in similar patients.

## Conclusions

This case illustrates the deadly synergy of DKA, hypercoagulability, and pancreatic cancer. The patient’s extensive thrombosis, confirmed by imaging and autopsy, aligns with the known prothrombotic nature of pancreatic malignancy. Her sudden deterioration emphasizes the importance of early VTE detection and anticoagulation, balanced against bleeding risks, in managing such complex cases. Clinicians should exercise caution with invasive procedures in patients with significant comorbidities and thrombotic predispositions.
